# Sewage Sludge Microbial Structures and Relations to Their Sources, Treatments, and Chemical Attributes

**DOI:** 10.3389/fmicb.2018.01462

**Published:** 2018-07-03

**Authors:** Altina Lacerda Nascimento, Adijailton Jose Souza, Pedro Avelino Maia Andrade, Fernando Dini Andreote, Aline Renée Coscione, Fernando Carvalho Oliveira, Jussara Borges Regitano

**Affiliations:** ^1^Department of Soil Science, “Luiz de Queiroz” College of Agriculture, University of São Paulo, Piracicaba, Brazil; ^2^Center of Soil and Environmental Resources, Agronomic Institute of Campinas, Campinas, Brazil; ^3^Biossolo Agricultura e Ambiente Ltda., Piracicaba, Brazil

**Keywords:** bacteria, wastewater, biological treatment, liming, molecular biology

## Abstract

Sewage sludges generation and their disposal have become one of the greatest challenges of the 21st century. They have great microbial diversity that may impact wastewater treatment plant (WWTP) efficiency and soil quality whether used as fertilizers. Therefore, this research aimed to characterize microbial community diversity and structure of 19 sewage sludges from São Paulo, Brazil, as well as to draw their relations to sludge sources [domestic and mixed (domestic+industrial)], biological treatments (redox conditions and liming), and chemical attributes, using molecular biology as a tool. All sludges revealed high bacterial diversity, but their sources and redox operating conditions as well as liming did not consistently affect bacterial community structures. *Proteobacteria* was the dominant phylum followed by *Bacteroidetes* and *Firmicutes*; whereas *Clostridium* was the dominant genus followed by *Treponema*, *Propionibacterium*, *Syntrophus*, and *Desulfobulbus*. The sludge samples could be clustered into six groups (C1 to C6) according their microbial structure similarities. Very high pH (≥11.9) was the main sludge attribute segregating C6, that presented very distinct microbial structure from the others. Its most dominant genera were *Propionibacterium* > > *Comamonas* > *Brevundimonas* > *Methylobacterium* ∼*Stenotrophomonas* ∼*Cloacibacterium*. The other clusters’ dominant genera were *Clostridium* > > *Treponema* > *Desulfobulbus* ∼*Syntrophus.* Moreover, high Fe and S were important modulators of microbial structure in certain sludges undertaking anaerobic treatment and having relatively low N-Kj, B, and P contents (C5). However, high N-Kj, B, P, and low Fe and Al contents were typical of domestic, unlimed, and aerobically treated sludges (C1). In general, heavy metals had little impact on microbial community structure of the sludges. However, our sludges shared a common core of 77 bacteria, mostly *Clostridium*, *Treponema*, *Syntrophus*, and *Comamonas*. They should dictate microbial functioning within WWTPs, except by SS12 and SS13.

## Introduction

Urban centers fast growth and industrial activities intensification generate high volumes of effluents daily (Atashgahi et al., 2015), which are collected or discharged into the sewage network reaching wastewater treatment plants (WWTPs) (Shchegolkova et al., 2016). WWTPs comprehend efficient and low-cost processes to treat domestic and industrial effluents (Wen et al., 2015; Dezotti et al., 2017; Bassin et al., 2018). Among the treatments, the biological aims to degrade toxic organic compounds (petroleum derivatives, pharmaceutical compounds, and other xenobiotics) and reduce pathogenic organisms, mitigating effects on human health and environment (Seviour and Nielsen, 2010; Yang et al., 2011; Biswas and Turner, 2012; Xia et al., 2015). The residue (or by-product) of this activity, the sewage sludge, has great microbial diversity, which may vary depending on sewage origin, treatment condition (e.g., liming and redox conditions), industrial activity, among others.

Many factors may modulate microbial community structure within WWTPs, which may change from autotrophic to heterotrophic bacteria depending on effluent source, for example (Cydzik-Kwiatkowska and Zielińska, 2016). *Proteobacteria* phylum (21–65%) was predominant in municipal WWTPs (domestic sewage), mostly belonging to *Betaproteobacteria* that represents a class of microorganisms related to organic matter degradation and nutrient cycling. Other less dominant taxa were *Bacteroidetes*, *Acidobacteria*, and *Chloroflexi* (Nielsen et al., 2010; Wan et al., 2011; Wang et al., 2012). *Proteobacteria* was also abundant in industrial sewages that often have high concentrations of recalcitrant compounds originating from pharmaceutical industries, petroleum refineries, animal feed factories, and others (Ibarbalz et al., 2013; Ma et al., 2015). Biological treatment condition may be another important modulating factor. For example, microorganisms were most abundant in both anaerobic and anaerobic-aerobic than in aerobic system, but *Proteobacteria* was most abundant in aerobic whereas *Bacteroidetes* was most abundant in anaerobic bioreactors (Hu et al., 2012).

It is also clear that chemical attributes, such as pH and macronutrient contents (Tan et al., 2006; Ibarbalz et al., 2013; Gao et al., 2016; Meerburg et al., 2016); presence of toxic compounds, such as organic pollutants and heavy metals (Bettiol and Ghini, 2011; Balcom et al., 2016); and biological treatment (redox) conditions (Hu et al., 2012) can directly impact sludge bacterial community structure. In Brazil, sulfur oxidoreductive bacteria community were composed by 22 families, which could be clustered by sludge sources and chemical attributes, such as S, K, Zn, Mn, P, and N (Meyer et al., 2016).

Despite the relevance of the microorganisms, the literature in this field presents some shortcomings. First, there are several studies addressing sludge microbial community structure in WWTPs, but they often regard a small number of samples. Second, current knowledge was attained employing mainly laboratory bioreactors and pilot systems (Ahmed, 2012; Saia et al., 2016), but controlled operating conditions (temperature, aeration, and effluent flow) shorten microbial community diversity (Muszyński et al., 2013, 2015). Third, several studies used conventional techniques, but only 60–90% of bacteria population are cultivable. The emergence of molecular techniques allowed better characterization of microbial structure and function directly in the environment (Liaw et al., 2010; Tomazetto and Oliveira, 2013; Lee et al., 2015), as well as better description of microbial community ecological role (Dezotti et al., 2017). However, these information are still scarce under realistic conditions (Biswas and Turner, 2012; Bassin et al., 2018), and even more in tropical countries.

Therefore, this research work aimed to evaluate whether microbial community structure of several sewage sludges from São Paulo State, Brazil, is related to WWTP conditions, such as sewage source [domestic or mixed (domestic+industrial)], biological treatment (redox) conditions, liming, urbanization, and industrial activity; as well as to sludge chemical attributes. It would supply useful information about hygiene measures needed and/or potential contamination resulting from sludge application as soil amendment.

## Materials and Methods

### Samples Collection and Characterization

Sewage sludge samples were collected from 19 WWTPs of São Paulo State, Brazil. Five samples (SS6, SS7, SS11, SS12, and SS13) were collected from metropolitan area of São Paulo City, the most urbanized and industrialized region within São Paulo State, whereas the others were collected from other municipalities (**Table 1**). Sample collection was performed at the sludges dewatering points, as described by EPA SW-865.

**Table 1:** Sewage sludges sources and treatments as well as main chemical attributes.

Sample	Treatment	pH	C/N	Fe g kg^-1^	Al g kg^-1^	S g kg^-1^	N-Kj g kg^-1^	P g kg^-1^	B mg kg^-1^
SS01	Ae/D	8.1	6.9	19.9	7.0	11.8	61	16.8	12.4
SS02	Ae/D	7.4	7.7	13.7	10.8	7.9	44	10.2	16.1
SS03	Ae/D	6.5	6.7	4.4	6.7	8.7	58	14.8	22.4
SS04	Ae/D	7.9	7.0	20.9	22.8	15.5	54	17.7	3.5
SS05	Ae/D+L	7.3	6.3	4.5	5.5	8.1	61	13.6	19.6
SS06	Ae/M	8.1	8.8	22.8	22.4	18.4	39	15.0	7.8
SS07	Ae/M	8.0	7.9	42.4	18.4	36.9	40	10.6	14.9
SS08	Ae/M	8.0	6.9	14.8	9.7	14.9	60	9.9	6.4
SS09	Ae/M	7.9	13.2	21.6	18.9	24.9	27	11.0	11.4
SS10	Ae/M	7.2	10.5	24.2	18.3	32.6	38	8.5	7.5
SS11	Ae/M+L	11.1	7.2	84.6	8.2	10.7	28	16.4	15.7
SS12	Ae/M+L	13.1	6.6	29.9	6.8	5.7	44	10.6	3.3
SS13	Ae/M+L	11.9	12.9	38.0	21.1	14.1	17	9.5	6.6
SS14	An/D	8.0	11.2	18.3	13.6	19.3	25	7.7	4.7
SS15	An/M	8.7	10.9	86.8	15.9	17.3	34	13.9	4.1
SS16	AeAn/D	7.9	7.6	22.5	48.5	7.9	35	14.3	1.7
SS17	AeAn/M	8.1	10.5	28.4	18.4	28.7	30	7.6	2.4
SS18	AeAn/M	7.9	8.6	12.4	19.8	19.9	40	11.9	7.4
SS19	AnAe/D+L	6.9	6.3	10.8	10.2	13.4	60	20.5	11.5
Mean		8.4	8.6	27.4	15.9	16.7	42	12.7	9.4

*Ae, aerobic; An, anaerobic; D, domestic; M, mixed (domestic+industrial); L, liming.*

For this purpose, three samples were collected from each WWTP. Each sample was composed of five subsamples (200 g) taken in 10-min intervals, mixed, and properly homogenized. They were conditioned in glass bottles and refrigerated until analysis according CONAMA Resolution 375/2006 (BRASIL, 2006).

### Chemical Attributes of the Samples

Moisture was determined according to EPA-SW 846. For this, sludge samples of 100 g were oven dried at 65°C, for 48 h. pH was measured using 2 g of moist sample and 20 ml of deionized water, which was stirred for 5 min at 220 rpm and rested for 30 min. For total inorganic N, 5 g of moist samples were distilled with 50 ml of 1.0 mol L^-1^ KCl, 0.2 g of MgO, and 0.2 g of Devarda alloy, which were taken in 5 mL of 20 g L^-1^ H_3_BO_3_ and titrated with 0.0025 mol L^-1^ H_2_SO_4_ (Bremner, 1996). Nitrite and nitrate were determined according to Mulvaney (1996). For organic N (N-Kj), 0.05 g of oven dried samples were mixed with 3 mL of concentrated H_2_SO_4_, placed in a digester block ( ± 360° C) for 3 h, distilled with 20 mL of 10 mol L^-1^ NaOH, which were also taken in 20 mL of 20 g L^-1^ H_3_BO_3_ and then titrated with 0.0025 mol L^-1^H_2_SO_4_ (American Public Health Association [APHA], 2005). Organic carbon (OC) was determined by the K_2_Cr_2_O_7_ method (Nelson and Sommers, 1996). Ca, K, P, Mg, S, Cu, Fe, Ni, Mn, Mo, Si, Zn, Al, As, Ba, Cd, Cr, Pb, Hg, and Na were extracted in microwave oven, according to EPA (2007). K and Na were quantified by flame photometry and the other elements by inductively coupled plasma atomic emission spectrometry (ICP-OES).

### Total DNA Extraction and Sequencing From Sludge Samples

For total DNA, 0.4 g of each sewage sludge sample was extracted individually using MoBio Power Soil DNA Isolation Kit (MoBio, United States), according to manufacturer’s instructions. Integrity of the extracted DNA was checked by electrophoresis (1% agarose gel), which was stained with ethidium bromide and visualized under ultraviolet light.

DNA sequencing was performed by Illumina MiSeq platform and library preparation based on Nextera XT index kit (Illumina, United States), targeting the V4 region of the 16S rRNA gene. This was amplified using a mixture of *4-Forward* and *4-Reverse primers* with pre-adapters (Supplementary Table S1). For the PCR reaction (final volume of 25 μL), 3.0 μL of PCR Buffer, 2.5 μL of MgCl_2_ (50 mM), 2.0 μL of DNTPs (2.5 mM), 0.1 μL of each primer mix, 0.3 μL Taq DNA polymerase (0.05 U/μL), 16 μL mili-Q water and 1.0 μL template DNA were utilized. Amplification conditions involved initial denaturation at 95°C for 3 min, 30 cycles at 95°C for 45 s, 57°C for 1 min: 45 s; 72°C for 1 min; followed by a final extension at 72°C for 4 min (Caporaso et al., 2011). PCR products were confirmed by electrophoresis in agarose gel (1%) and resulted in amplified fragments of ∼430 bp. Amplified DNA was then purified with QiaQuick PCR kit, quantified by spectrophotometry (ND-1000), and PCR products stored (-20°C) for sequencing.

After DNA purification, another PCR reaction was performed to bind adapters (an index pair) to identify sequence origin. This consisted of 3.0 μL of PCR buffer, 2.5 μL of MgCl_2_ (50 mM), 2.0 μL of DNTPs (2.5 mM), 5 μL of each adapter (index), 0.3 μL of Taq DNA polymerase (0.05 U/μL), 17.2 μL of mili-Q water, and 15 μL of previous reaction product (final volume = 50 μL). Amplification conditions consisted of 95°C for 3 min, five cycles at 95°C for 45 s, 57°C for 1 min: 45 s; 72°C for 1 min; followed by a final extension at 72°C for 4 min. Sequencing was carried out at the University of São Paulo (USP/ESALQ), by the Animal Biotechnology Laboratory within the Animal Science Department.

### Bioinformatic and Statistical Analyses

Quantitative Insights into Microbial Ecology (QIIME) program was used for DNA sequencing analysis (Caporaso et al., 2010). Sequences quality was set at 20. Removal of poor quality sequences, primers, barcodes, and adapters were performed with CLC Genomics Workbench 6 (CLCbio). Operational taxonomic units (OTUs) were grouped in 3% distance level (97% of similarity) and classification was performed by the Ribosomal Database Project (RDP Classifier). OTUs were also used to estimate ecological parameters using Chao 1, Simpson, and Shannon diversity indexes. Clustering of the samples was performed by principal coordinate analysis (PCoA) (Ramette, 2007), and tested by similarity analysis (ANOSIM) on Past^®^ software (v.3.2) (Hammer et al., 2001). ANOSIM was also used to verify sample similarities according sludge sources, biological (redox) treatments, and liming.

Relationship between bacterial community composition and sludges sources, treatments, and chemical attributes (pH, moisture, N-NH_4_^+^, N-NO_2_^-^/NO_3_^-^, organic N (N-Kjeldahl = N-Kj), organic carbon (OC), K, Ca, Fe, P, S, Mg, Na, Cd, Cr, Cu, Hg, Mn, Mo, Ni, Pb, Se, Zn, Al, As, and Ba) were settled by redundancy analysis (RDA) on Canoco^®^ software (v.4.5). Graphics were plotted on Origin^®^ software (v.10.5), but heatmap graphical scales were built in R software, using “gplots” and “RColorBrewer” packages^1^.

## Results

### Sewage Sludges Locations, Treatments, Sources, and Main Chemical Attributes

Samples identification and main chemical attributes affecting microbial community and their clustering were presented in **Table 1**. The other chemical attributes were summarized from a previous thesis work (Nascimento, 2015) and presented as supplementary material (Supplementary Table S2). Thirteen samples underwent aerobic (SS1 to SS13) whereas the other six (SS14-SS19) underwent either strictly anaerobic or combined aerobic-anaerobic treatments during biological digestion. Eight samples were collected from domestic (SS1-SS5, SS14, SS16, and SS19) whereas the others were collected from mixed sewers. Only five samples were limed (SS5, SS11, SS12, SS13, and SS19).

### Structure and Composition of Sewage Sludges Bacterial Communities

A total of 7,219,247 16S RNA gene sequences were attained. After removal of low quality sequences (cut level = 3%), OTUs matrixes showed that all sludges presented high diversity indexes (Supplementary Table S3). Although sequencing would contain inactive (dormant and dead) microorganisms, it should not impact diversity as verified by Liang et al. (2017).

**FIGURE 1:**
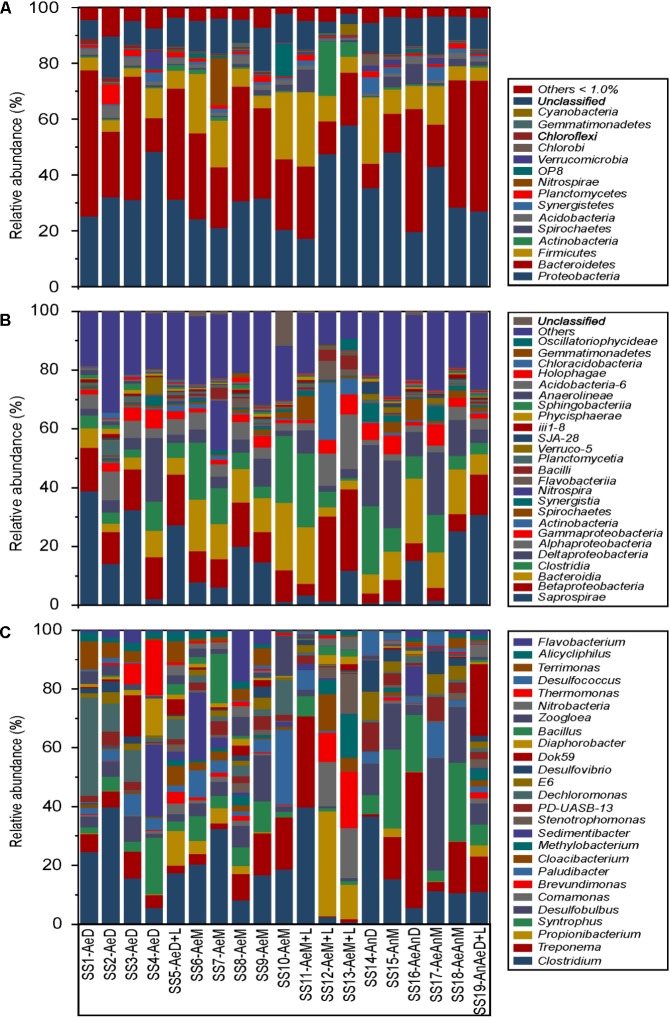
Relative microbial community abundance of 19 sewage sludges from São Paulo State, Brazil. (**A**, phyla; **B**, classes; **C**, genera; Others, members with relative abundance < 1%).

RDP Classifier identified 68 phyla, 164 classes, and 665 genera of bacteria. The most abundant phyla were *Proteobacteria* > *Bacteroidetes* > *Firmicutes*, corresponding to >73% of the DNA sequences (**Figure 1A**); whereas the most abundant classes were *Saprospirae* > *Betaproteobacteria* > *Bacteroidia* > *Clostridia* > *Deltaproteobacteria* (**Figure 1B**). In addition, *Betaproteobacteria* was the most abundant class within the *Proteobacteria* phylum (∼37% of the sequences), followed by *Deltaproteobacteria* (∼26%), *Alphaproteobacteria* (∼16%), and *Gammaproteobacteria* (∼11%); whereas *Saprospirae* was the most abundant class within the *Bacteroidetes* (∼46%), followed by *Bacteroidia* (∼36) and *Flavobacteria* (∼3%) (**Figures 1A,B**). Within the Firmicutes, the most abundant classes were *Clostridia* (∼87%) and *Bacilli* (∼9%) (**Figures 1A,B**). Finally, the most abundant genera were *Clostridium* > *Treponema* > *Propionibacterium* > *Syntrophus* > *Desulfobulbus* > *Brevundimonas* > *Paludibacter* > *Cloaci-bacterium* > *Methylobacterium* (**Figure 1C**). Despite distinctions in sewage sources and treatments, their bacterial community presented a common core of 77 genera, being *Clostridium*, *Treponema*, *Syntrophus*, and *Comamonas* the most abundant ones (Supplementary Figure S1).

### Clusters and Relations With Sludge Sources, Treatments, and Chemical Attributes

**FIGURE 2:**
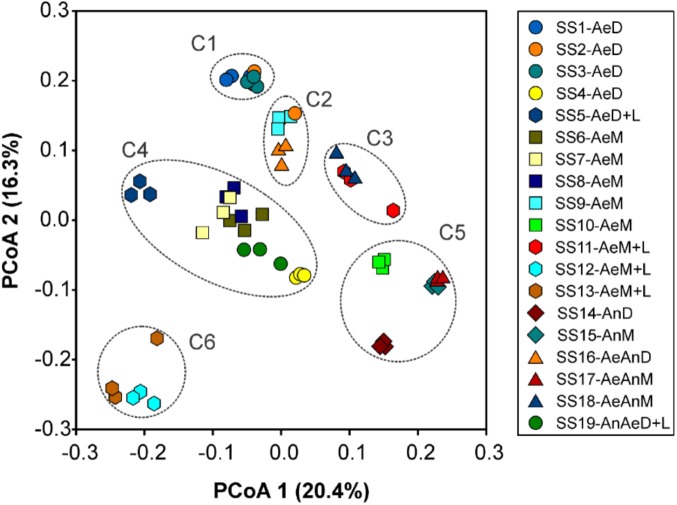
Principal coordinate analysis (PCoA) for bacterial community structure of 19 sewage sludges from São Paulo State, Brazil (*n* = 3). Axis values indicated percentage of variance.

The sludge samples could be grouped in six clusters according to PCoA: C1 (SS1, SS2, and SS3), C2 (SS9 and SS16), C3 (SS11 and SS18), C4 (SS4, SS5, SS6, SS7, SS8, and SS19), C5 (SS10, SS14, SS15, and SS17), C6 (SS12 and SS13). Its main two coordinates explained 36.7% of sludges’ bacterial community structures (**Figure 2**). This result was also validated by similarity analysis of their bacterial communities (**Table 2**).

**Table 2:** Analysis of similarity (ANOSIM) for microbial community structure and clusters formation for 19 sewage sludges from São Paulo State, Brazil.

	*R_value_*					
Clusters	C1	C2	C3	C4	C5	C6
C1	0.00	0.33	0.69^∗^	0.50^∗^	0.96^∗^	1.00^∗^
C2	–	0.00	0.41	0.09	0.81^∗^	1.00
C3	–	–	0.00	0.26	0.38	1.00^∗^
C4	–	–	–	0.00	0.53^∗^	0.58^∗^
C5	–	–	–	–	0.00	0.99^∗^
C6	–	–	–	–	–	0.00


*Based on the OTUs matrix attained by 16S rRNA sequencing (Illumina platform). C1, SS1, SS2, and SS3; C2, SS9 and SS16; C3, SS11 and SS18; C4, SS4, SS5, SS6, SS7, SS8, and SS19; C5, SS10, SS14, SS15, and SS17; C6, SS12 and SS13. R_value_ = degree of similarity; R_value_ > 0.75 means that samples differed; 0.50 > R_value_ > 0.75 means that samples overlapped; and R_value_ < 0.50 means that samples did not differ. ^∗^P_value_ < 0.002.*

**FIGURE 3:**
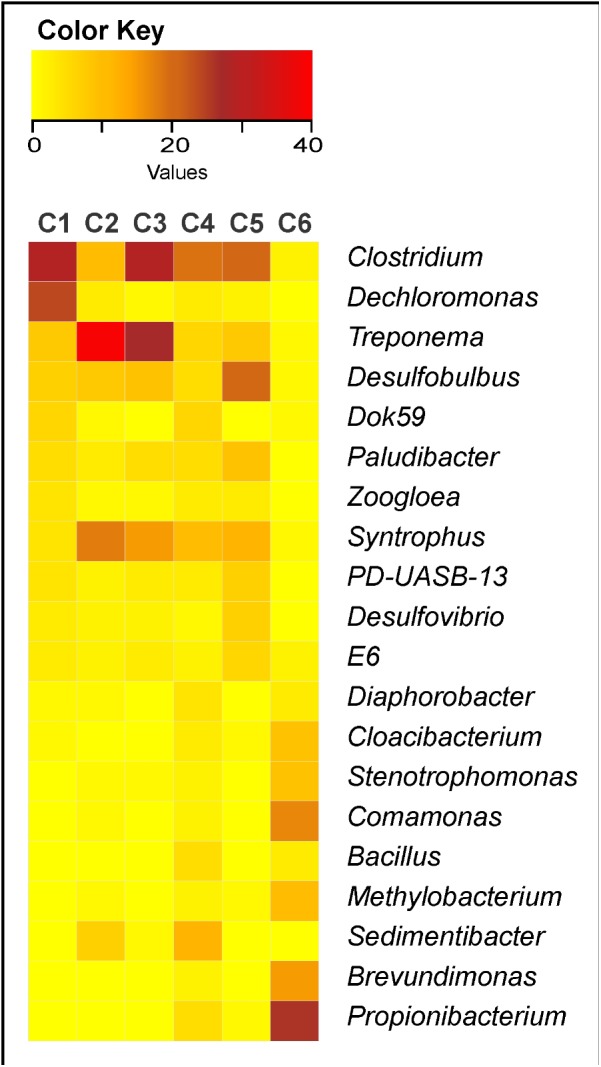
Heatmap of the 20 most abundant bacterial genera found in 19 sewage sludges from São Paulo State, Brazil. C1, cluster 1 (*n* = 3 samples); C2, cluster 2 (*n* = 2 samples); C3, cluster 3 (*n* = 2 samples); C4, cluster 4 (*n* = 6 samples); C5, cluster 5 (*n* = 4 samples); and C6, cluster 6 (*n* = 2 samples). Red color, most abundant genus; Yellow, least abundant genus.

C6 showed bacterial community very distinct from the others, with relative dominance of *Propionibacterium*, *Comamonas*, *Brevundimonas*, *Methylobacterium*, *Stenotrophomonas*, and *Cloacibacterium* (**Figure 3**). The other clusters (C1-C5) generally presented great abundance of *Clostridium*, *Treponema*, *Syntrophus*, and *Desulfobulbus*, except that C1 showed low abundance of *Syntrophus* and high abundance of *Dechloromonas*; C2 showed relative high abundance of *Sedimentibacter*; C3 showed relative high abundance of *Paludibacter*; C4 showed relative high abundance of *Sedimentibacter* and also of *Dok59* and *Bacillus*; and C5 showed relative high abundance of *Paludibacter*, *PD-UASB-13*, *Desulfovibrio*, and *E6* (**Figure 3**).

**FIGURE 4:**
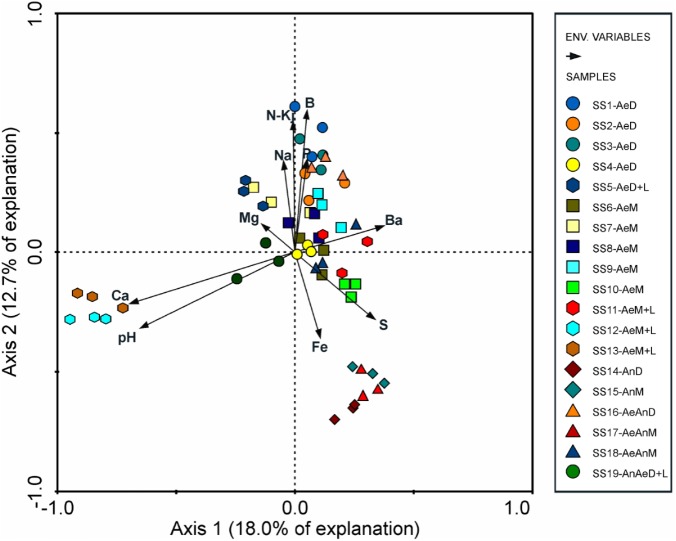
Redundancy analysis (RDA) between chemical attributes and bacterial community of 19 sewage sludges from São Paulo State, Brazil (*n* = 3 replicates).

Sewage sources (domestic or mixed) and biological treatments (redox conditions) did not affect consistently the bacterial community structuring (**Table 3**), suggesting that clusters were formed due to other factors, likely related with sludges chemical attributes as suggested by RDA (**Figure 4**). In fact, pH (λ = 0.11, *P*-value < 0.002), Fe (λ = 0.07, *P*-value < 0.002); B and Mg (λ = 0.06, *P*-value < 0.002); Na (λ = 0.05, *P*-value < 0.002); and P, Ba, organic N (N-Kj), and Ca (λ = 0.04, *P*-value < 0.002) contents were the sludge attributes most related to microbial community structuring and clustering; whereas organic carbon (OC), inorganic N (in the different forms), Hg, Se, and As contents, C/N ratio, and moisture were not correlated with sludges bacterial community structures (λ < 0.01 and *P*-value > 0.05) (Supplementary Table S2).

**Table 3:** Analysis of similarity (ANOSIM) for microbial community structure as affected by sources and treatments of 19 sewage sludges from São Paulo State, Brazil.

Sludge treatment	R*_value_*		Ae	AeAn	AnAe
	Ae		000	0.0631	0.2454^∗^
	Ae-An		–	000	0.3214^∗^
	An-Ae		–	–	000

**Sludge source**	**R*_value_***	**D**	**M**	**D+L**	**M+L**

	D	000	0.1406	0.2507	0.3179^∗^
	M	–	000	0.1490	0.4504^∗^
	D+L	–	–	000	0.1736
	M+L	–	–	–	000

*Based on the OTUs matrix attained by 16S rRNA sequencing (Illumina platform). Ae, aerobic; AeAn, aerobic then anaerobic; AnAe, anaerobic then aerobic; D, domestic; M, mixed (domestic+industrial); D+L, domestic with liming; M+L, mixed with liming; R_value_, degree of similarity; R_value_ > 0.75 means that samples differed; 0.50 > R_value_ > 0.75 means that samples overlapped; and R_value_ < 0.50 means that samples did not differ. ^∗^P_value_ < 0.002.*

## Discussion

### Structure and Composition of Sewage Sludges Bacterial Communities

WWTPs bacterial community exhibited low variation at higher taxonomical levels (e.g., phylum) even for distinct geographic regions and sludge treatments (Philippot et al., 2010; Ibarbalz et al., 2013; Hatamoto et al., 2017) (**Figure 1A**). In all samples, independently of sewer operating condition, the most abundant phyla were *Proteobacteria* > *Bacteroidetes* > *Firmicutes* (**Figure 1A**). Similar results were reported for sludges from China (Zhang et al., 2012; Shu et al., 2015b; Gao et al., 2016; Liang et al., 2017). However, the literature shows some contrasting results. Meerbergen et al. (2017) found predominantly *Proteobacteria*, *Bacteroidetes*, and *Actinobacteria* for domestic sludges, but *Planctomycetes*, *Chloroflexi*, *Acidobacteria*, and *Chlorobi* for industrial sludges. *Proteobacteria* usually predominated in domestic sewage sludges, corresponding from 30 to 65% of the total sequences (Liang et al., 2017; Meerbergen et al., 2017), as well as in various other environments, such as soil (Roesch et al., 2007; Spain et al., 2009; Sun et al., 2015) and rhizosphere (Jiang et al., 2016). *Proteobacteria* usually presented wide diversity and metabolic capacity, acting in important environmental functions such as the cycles of C, N, S, and P (Friedrich et al., 2005; Meyer et al., 2016). *Bacteroidetes* were often reported as proteolytic bacteria, involved in degrading protein to volatile phenolic acids and ammonia (NH_3_) (Yi et al., 2014). Their abundance was correlated with total solid contents when submitted to anaerobiosis (Liu et al., 2016). *Firmicutes* were often widely distributed in anaerobic sludge treatment systems (Yang et al., 2014) and were versatile in degrading a vast array of environmental substrates (Liu et al., 2016). They may act on metabolic pathways responsible for producing volatile fatty acids, which can be used by other microbial groups.

The most abundant classes were *Saprospirae* > *Betaproteo-bacteria* > *Bacteroidia* > *Clostridia* > *Deltaproteobacteria* > *Alphaproteobacteria* > *Gammaproteobacteria* > *Actinobacteria* > *Spirochaetes* (**Figure 1B**). Liang et al. (2017) and Shu et al. (2015a) reported relative high abundance of *Betaproteobacteria*, which is often associated with organic matter degradation and S cycle (Friedrich et al., 2005; Takai et al., 2005). In Denmark, however, several studies reported low occurrence of *Saprospirae* in full scale WWTPs (Nielsen et al., 2012; Kong et al., 2007; Muszyński et al., 2015). The members of this class were predominant in marine environments, but could also be found in fresh water and sewage sludges degrading complex organic compounds (Nielsen and McMahon, 2014).

Lower taxonomic levels (e.g., genus) showed higher bacterial community differentiation among WWTPs (**Figure 1C**), corroborating with the literature (Philippot et al., 2010; Ibarbalz et al., 2013). The most abundant genera were *Clostridium* > *Treponema* > *Propionibacterium* > *Propionibacterium* > *Syntrophus* > *Desulfobulbus* > *Comamonas* > *Brevundimonas* > *Paludibacter* > *Cloacibacterium* > *Methylobacterium* > *Sedimentibacter* > *Stenotrophomonas* (**Figure 1C**). A great diversity of bacterial genera were also described in the literature (Lee et al., 2015), which several times differed from ours (Ibarbalz et al., 2013; Stiborova et al., 2015; Gao et al., 2016). It could be explained by the fact that WWTPs comprise open and very dynamic systems allowing rapid succession among microbial community members during spatial and temporal scales (Shu et al., 2015a). Nevertheless, our samples showed a common nucleus of 77 bacteria, represented mostly by *Clostridium*, *Treponema*, *Syntrophus*, and *Comamonas* (Supplementary Figure S1). Gao et al. (2016) identified a common nucleus of 177 genera for sewage sludges from China. This shared core of bacteria is usually responsible for the main functions in the environment (Shu et al., 2015b). Several pathogenic bacteria, such as *Clostridium*, *Treponema*, *Stenotrophomonas*, *Bacillus*, *Mycobacterium*, and *Acinetobacter* were also identified, in accordance to Stiborova et al. (2015).

### Clusters and Relations With Sludge Sources, Treatments, and Chemical Attributes

Sewage sources (domestic or mixed) and biological treatments (redox conditions) did not affect microbial community structure (**Table 3**) and diversity (Chao1, Simpson and Shannon) (Supplementary Table S3), at least not consistently, similarly to Hai et al. (2014). However, Gao et al. (2016) found that biological treatment (redox conditions) influenced microbial community, which was more diverse in aerobic tanks; whereas Meerburg et al. (2016) reported structural differences in the bacterial community of domestic and industrial sludges. Gao et al. (2016) found a common core of 177 bacteria genera to their samples and we found a common core of 77 bacteria that corresponded to 85% of the identified sequences. They considered only seven samples from strictly domestic sewers that would explain their greater similarity. Normally, bacterial communities of domestic sewers are more diverse due to its larger fraction of readily degradable organic material (Meerbergen et al., 2017). Industrial sewers receive recurrent discharges of more recalcitrant and toxic pollutants (Gao et al., 2016), such as heavy metals and antimicrobial agents (Bettiol and Ghini, 2011; Balcom et al., 2016), thus limiting microbial diversity. Hu et al. (2012) reported high similarity between bacterial communities of five sludges from China, whereas Zhao et al. (2014) reported substantial disparity, mainly due to their spatial variation and biological composition. Meyer et al. (2016) also reported significant variation in the structure of S oxidoreductive bacteria from south Brazil.

On the other side, certain chemical attributes showed direct connections to sludge microbial community structures (**Figure 4** and Supplementary Table S2), favoring samples segregation in clusters (**Figure 2** and **Table 2**). High pH values (≥11.9) resulted from liming were responsible for segregating C6 (SS12 and SS13) and enhancing Ca contents (**Figure 4**). Its most abundant phyla were *Actinobacteria*, *Proteobacteria*, and *Bacteroidetes*; whereas the most abundant genera were *Propionibacterium*, *Comamonas*, *Brevundimonas*, *Methylobacterium*, *Stenotrophomonas*, and *Cloacibacterium* (**Figure 3**). Other limed samples (SS5, SS11, and SS19) presented lower pH (**Table 1**) and; therefore, very distinct microbial structure from C6. Despite having similar operating conditions as SS12 and SS13, SS11 also showed slightly lower pH as well as lower Cu and Zn and higher Fe and Pb contents (**Table 1**). It has been demonstrated that 1 pH-unit may considerably affect bacterial community structure and composition (Fierer and Jackson, 2006). Gao et al. (2016) also observed distinct phylogeny (*Proteobacteria*, *Bacteroidetes*, *Acidobacteria*, *Chloroflexi*, and *Firmicute*s) at lower pH values (∼8.0). Liming to high pH values usually decreases microbial community diversity (Blaszczyk and Krzysko-Lupicka, 2013; Farzadkia and Bazrafshan, 2014), being an important tool promoting sludge hygienization (i.e., pathogens control). In our case, high pH did not affect microbial diversity (Supplementary Table S3) but affected its structure inclusive favoring extremotolerant bacterial groups, such as *Actinobacteria* (**Figure 1A**). Several studies showed that pH affected microbial community diversity and composition in soils (Rousk et al., 2009; Cho et al., 2016; Wu et al., 2016) and sewage sludges (Maspolim et al., 2015). Lauber et al. (2009) found that bacterial phyla (*Acidobacteria*, *Actinobacteria*, *Bacteroidetes*, and α, β, and γ-*Proteobacteria*) relative abundance did not depend on sludge location, but on pH instead. Therefore, pH may modulate microbial community by controlling nutrients availability and enzymatic processes that are essential to microbial metabolism (Fierer and Jackson, 2006; Madigan et al., 2016).

C5 (SS10, SS14, SS15, and SS17) segregation was related mostly to Fe but also to S, B, P, and N-kj, and contents (**Figure 4**). These samples underwent anaerobic treatment, except for SS10 (**Table 1**). Under anaerobiosis, both Fe and S have important roles in redox reactions (Ma et al., 2014), acting as final electron acceptors (Moreira and Siqueira, 2006; Shrestha et al., 2009; Alexandre et al., 2012). Fe is reduced to its most soluble form (Fe^3+^ → Fe^2+^) (Shrestha et al., 2009) and reducing bacteria are important mediators of C and N transformations (Tan et al., 2006; Wang et al., 2009; Ding et al., 2014). Several bacteria associated with Fe reduction were identified, such as *Acidithiobacillus*, *Ferrimicrobium*, and *Nitrospira* (**Figure 1B**). In parallel, S reduction generates energy in anaerobic environments (Aida et al., 2014); and it is crucial in structuring microbial community. It could be ratified by relative high abundance of *Desulfobulbus* and presence of *Desulfovibrio* as well as *Desulfococcus*, *Desulforhabdus*, and *Desulfovirga* in the samples (**Figure 1C**). *Desulfovibrio*, *Desulforhabdus*, and *Smithella* were very efficient in removing S from anaerobically treated sludges (Aida et al., 2015). SS10 was the only sample having aerobic treatment and showed the highest S concentration (**Table 1**). C5 samples had lower N-Kj likely due to denitrification (Yamashita and Yamamoto-Ikemoto, 2014), resulting sludges with slightly higher C/N ratios (10.8 versus 8.0) (**Table 1**). Systems operated initially under anaerobic followed by aerobic conditions usually contribute most to N loss since they warranty anoxic denitrification and aerobic nitrification, thus converting ammonia (NH_4_^+^) to gaseous N (N_2_, NO_2_, and N_2_O) (Ruiz et al., 2006; Kassab et al., 2010; Yao et al., 2013a,b; Zhang et al., 2014). These samples also showed low P and B contents (**Table 1**).

All C1 samples (SS1, SS2, and SS3) derived from domestic sewers, aerobically treated and without liming (**Table 1**). They presented higher B, P, and N-Kj as would be expected from their higher organic matter pool, thus generating sludges with lower C/N ratios (**Table 1**). They also presented high Na and low Fe and Al contents (**Table 1**), which would be expected by their source nature (domestic). The most abundant genera were *Clostridium* > *Dechloromonas* >> *Treponema* > *Desulfobulbus* > *Dok59* (**Figure 3**). Likewise, *Clostridium*, *Treponema*, and *Desulfobulbus* were also abundant in C2 and C3 (**Figure 3**). High abundance of *Clostridium* in domestic sludges was expected since it represents 10–40 % of human intestinal microbiota (Manson et al., 2008; Lopetuso et al., 2013). *Clostridium* was usually the most abundant genera in activated sludges, whereas *Desulfobulbus* and *Dechloromonas* were often associated with nutrients (such as N and S) removal from WWTPs (Aida et al., 2015).

Toxic inorganic elements, such as heavy metals (excluding the micronutrients), did not impact microbial community structure (Supplementary Table S2 and **Figure 4**). These element contents were below those set by the Brazilian legislation for sludge use in agriculture (CONAMA 375/2006). Only one sample (SS7) exceeded threshold concentration for Ni and three (SS1, SS12, and SS13) for Zn, but both are plant micronutrients. On the other side, Cd, Cr, and Ag inhibited important microorganisms for biological treatment, thus impacting sludge bacterial community (Wells et al., 2011).

## Conclusion

All sewage sludges presented high bacterial diversity. Their sources and biological treatment (redox) conditions did not consistently affect bacterial community structures. Overall, *Proteobacteria* was the dominant phylum, followed by *Bacteroidetes* and *Firmicutes*. Their predominant classes were *Betaproteobacteria* (∼37%), *Saprospirae* (∼46%), and *Clostridia* (∼87%), respectively. *Clostridium* was the dominant genera, followed by *Treponema*, *Propionibacterium*, *Syntrophus*, and *Desulfobulbus*. Moreover, the samples were clustered into six groups according similarity of microbial community structures, which were related to their chemical attributes. High pH values (≥11.9) resulted from liming impacted mostly bacterial community structures and segregated C6, in which predominated *Propionibacterium*, *Comamonas*, *Brevundimonas*, *Methylobacterium*, and *Cloacibacterium* that are extremotolerant organisms. However, *Clostridium*, *Treponema*, *Desulfobulbus*, *and Syntrophus* were usually the most abundant ones in the other clusters, except that C1 presented relatively high abundance of *Dechloromonas*; C2 and C4 presented relatively high abundance of *Sedimentibacter*, and C3 and C5 presented relatively high abundance of *Paludibacter*. High Fe and S contents were important modulators of microbial structure for certain sludges undertaking anaerobic treatments and having relatively low N-kj, B, and P contents (C5); whereas high N-Kj, B, and P contents were important modulator for domestic, aerobically treated, and unlimed sludges having low Fe and Al contents (C1). Toxic inorganic elements, such as heavy metals (excluding micronutrients), had little impact on microbial community structure of the sludges. Nevertheless, the sludges shared a common core of 77 bacteria, being *Clostridium*, *Treponema*, *Syntrophus*, and *Comamonas* the most abundant ones.

## Author Contributions

AN contributed to sample collection and processing as well as chemical and microbiological analyses. AS contributed to overall data analyses and manuscript writing. PA performed bioinformatic analyses. FA contributed to research idealization and manuscript revision. AC performed chemical attributes analyses. FO contributed to sample collection and research idealization. JR idealized, wrote, and revised the manuscript and coordinated the research.

## Conflict of Interest Statement

The authors declare that the research was conducted in the absence of any commercial or financial relationships that could be construed as a potential conflict of interest.

## Footnotes

1 https://www.r-project.org
