# Supplementary material for: Sewage Sludge Microbial Structures and Relations to Their Sources, Treatments, and Chemical Attributes
Source: Front Microbiol. 2018 Jul 3;9:1462. doi: 10.3389/fmicb.2018.01462 (PMC6037839; doi:10.3389/fmicb.2018.01462)
Supplement: Supplementary file 1 [file Presentation_1.pdf]

Sewage sludge microbial structures and relations to their sources, treatments, and chemical attributes

Altina Lacerda Nascimento[1]; Adijailton Jose de Souza[1]; Pedro Avelino Maia de Andrade[1]. Fernando Dini Andreote[1]; Aline Renée Coscione[2]. Fernando Carvalho Oliveira[3]; Jussara Borges Regitano[1.3]

[1]*Department of Soil Science, "Luiz de Queiroz" College of Agriculture, University of São Paulo. Piracicaba. Brazil*

[2]*Center of Soil and Environmental Resources, Agronomic Institute of Campinas, Campinas, Brazil*

[3]*Biossolo Agricultura e Ambiente LTDA, Piracicaba, Brazil.*

[4]*Corresponding author:* regitano@usp.br

Supplementary Figure S1. Relative abundance of common genera to 19 sewage sludges from São Paulo State, Brazil. *Others = members with relative abundance lower than 1%.

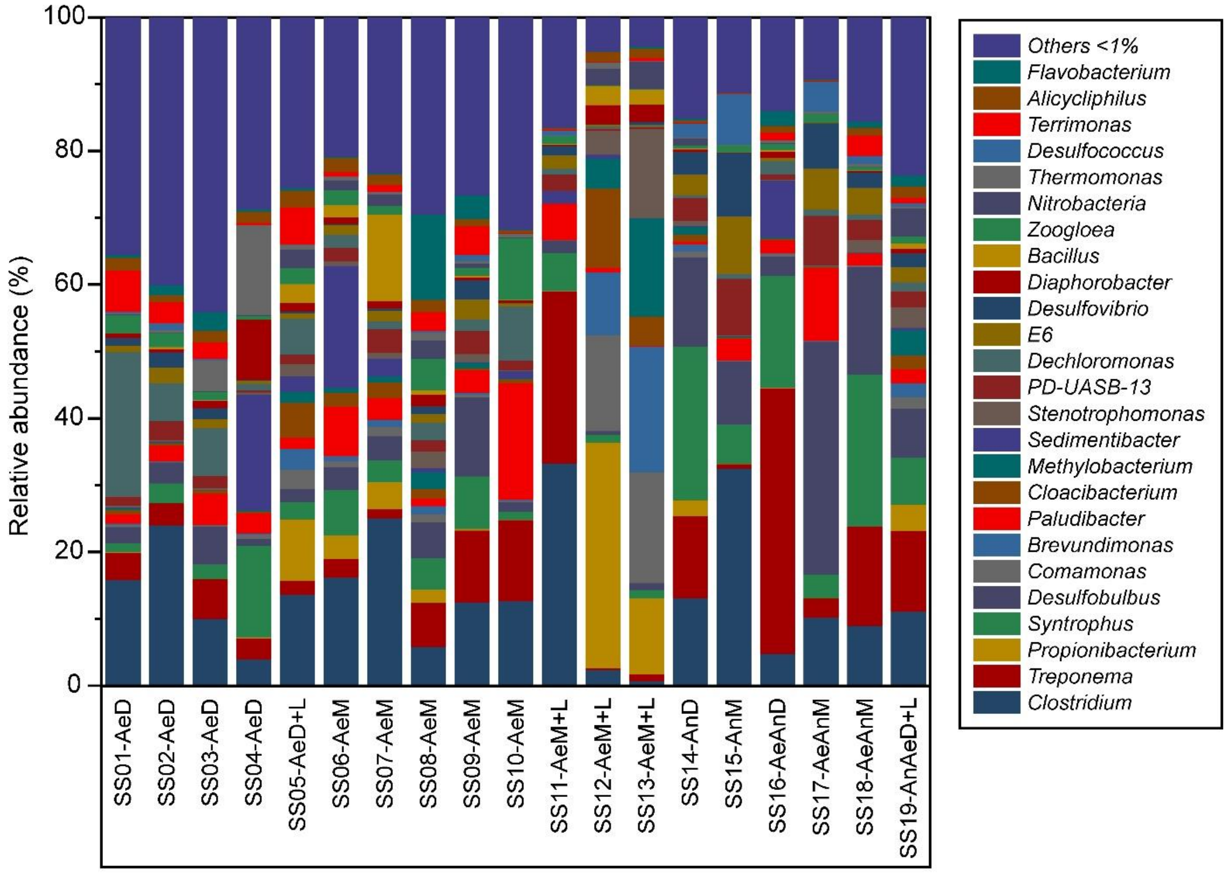

Supplementary Table 1. Primers mix used in PCR reaction for the 16S rRNA gene sequencing.

**Primers**	**Sequence 5' → 3'**
16SV4FPCR1_1 F	5'TCGTCGGCAGCGTCAGATGTGTATAAGAGACAGAYTGGGYDTAAAGNG
16SV4FPCR1_2 F	5'TCGTCGGCAGCGTCAGATGTGTATAAGAGACAGNAYTGGGYDTAAAGNG
16SV4FPCR1_3 F	5'TCGTCGGCAGCGTCAGATGTGTATAAGAGACAGNNAYTGGGYDTAAAGNG
16SV4FPCR1_4 F	5'TCGTCGGCAGCGTCAGATGTGTATAAGAGACAGNNNAYTGGGYDTAAAGNG
16SV4RPCR1_1 R	5'GTCTCGTGGGCTCGGAGATGTGTATAAGAGACAGCCGTCAATTCMTTTRAGT
16SV4RPCR1_2 R	5'GTCTCGTGGGCTCGGAGATGTGTATAAGAGACAGNCCGTCAATTCMTTTRAGT
16SV4RPCR1_3 R	5'GTCTCGTGGGCTCGGAGATGTGTATAAGAGACAGNNCCGTCAATTCMTTTRAGT
16SV4RPCR1_4 R	5'GTCTCGTGGGCTCGGAGATGTGTATAAGAGACAGNNNCCGTCAATTCMTTTRAGT

Supplementary Table 2. Summary of the chemical attributes from 19 sewage sludges from São Paulo State, Brazil and their contribution (**λ)** to microbial community structures according redundancy analysis (RDA).

**Attribute**		**Range**	**Mean**	**Median**	**Lambda (λ)**	**P_{value}**
pH		6.5 - 13.1	8.4	8.0	0.11	0.002
Fe	g kg^{-1}	4.4 - 86.8	27.4	21.6	0.07	0.002
B	mg kg^{-1}	1.7 - 22.4	9.4	7.4	0.06	0.002
Mg	g kg^{-1}	1.0 - 4.5	2.6	2.4	0.06	0.002
Na	g kg^{-1}	0.1 – 1.6	0.8	0.6	0.05	0.002
P	g kg^{-1}	7.6 - 20.5	12.7	11.9	0.04	0.002
Ca	g kg^{-1}	7.2 - 142.9	30.5	13.8	0.04	0.002
Ba	mg kg^{-1}	90 - 1082	471	411	0.04	0.002
N-Kj	g kg^{-1}	17 - 61	42	40	0.04	0.002
Cu	mg kg^{-1}	85 - 573	312	290	0.03	0.002
Al	g kg^{-1}	5.5 - 48.5	15.9	15.9	0.03	0.002
Ni	mg kg^{-1}	6.5 - 522.7	102.0	69.6	0.03	0.002
Mn	mg kg^{-1}	133 - 449	218	201	0.03	0.004
Zn	mg kg^{-1}	245 - 4592	1590	1309	0.02	0.002
Cr	mg kg^{-1}	16 - 593	206	104	0.02	0.004
$N\text{-}NH_4^+$	mg kg^{-1}	20 - 335	126	122	0.02	0.010
Mo	mg kg^{-1}	0.5 - 18.7	4.1	2.7	0.02	0.018
K	g kg^{-1}	0.5 - 4.6	1.5	1.1	0.02	0.094
$N\text{-}NO_2^-/NO_3^-$	mg kg^{-1}	2.2 – 7.2	4.1	3.8	0.02	0.112
S	g kg^{-1}	5.7 - 36.9	16.7	14.9	0.01	0.010
Pb	mg kg^{-1}	16 - 119	41	33	0.01	0.010
C/N		6.3 - 13.2	8.6	7.7	0.01	0.144
Moisture	%	56 - 87	74	75	0.01	0.450
Hg	mg kg^{-1}	1.7 - 11.3	6.3	5.3	0.01	0.430
Cd	mg kg^{-1}	0.2 - 6.9	2.7	2.6	0.01	0.700
OC	g kg^{-1}	203 - 425	338	344	< 0.01	0.212
Se	mg kg^{-1}	< LQ - 11.0	3.1	2.6	< 0.01	0.226
As	mg kg^{-1}	1.9 - 10.7	6.9	7.0	< 0.01	0.624

Supplementary Table 3. Bacterial diversity indexes for 19 sewage sludges from São Paulo State, Brazil.

**Samples**	**Chao1**	**Simpson (1-D)**	**Shannon-Wiener (H)**
SS1	296	0.995	5.46
SS2	242	0.993	5.26
SS3	319	0.995	5.55
SS4	318	0.995	5.48
SS5	405	0.997	5.83
SS6	393	0.997	5.81
SS7	341	0.995	5.55
SS8	433	0.997	5.87
SS9	340	0.996	5.66
SS10	303	0.995	5.40
SS11	378	0.996	5.64
SS12	367	0.996	5.63
SS13	389	0.996	5.68
SS14	515	0.997	5.97
SS15	358	0.996	5.65
SS16	368	0.996	5.70
SS17	399	0.996	5.69
SS18	339	0.996	5.60
SS19	422	0.996	5.77
